# Radiological Detection of a Pulmonary Artery Pseudoaneurysm in Lung Adenocarcinoma: A Rare Association and a Case Report

**DOI:** 10.7759/cureus.82425

**Published:** 2025-04-17

**Authors:** Corey Mumaw, Yael Levy, Sarah Quinn, David Ratliff

**Affiliations:** 1 School of Medicine, Lake Erie College of Osteopathic Medicine, Bradenton, USA; 2 Department of Radiology, Baptist Health, Jacksonville, USA

**Keywords:** coil embolization, computed tomography, lung cancer, pseudoaneurysm, pulmonary artery pseudoaneurysm

## Abstract

Pulmonary artery pseudoaneurysm (PAP) is a rare and potentially life-threatening vascular abnormality that is commonly associated with infections, such as tuberculosis. However, their occurrence in the setting of lung adenocarcinoma is exceedingly rare. We report a case of a 67-year-old man with stage III non-small cell lung cancer (NSCLC) who had a pulmonary artery pseudoaneurysm incidentally detected on follow-up maintenance imaging. The pseudoaneurysm was contiguous with pulmonary artery branches in the right lower lobe and associated with a progressively enlarging necrotic tumor region, suggesting that vascular invasion contributed to its formation. The patient was not experiencing any complications or hemoptysis at the time of identification; however, the pseudoaneurysm was deemed at risk for rupture and was successfully treated with coil embolization. This case highlights the importance of closely monitoring for vascular abnormalities in malignancy and early intervention to prevent potentially fatal complications.

## Introduction

Pulmonary artery pseudoaneurysm (PAP) is a rare and life-threatening vascular abnormality characterized by the focal dilatation of the pulmonary artery wall. The incidence of PAPs has been estimated to be one in 14,000 [1]. They are most commonly found secondary to infections but have also been linked to malignancy in rare cases [2]. One of the most well-known causes is Rasmussen’s pseudoaneurysm, which normally arises in the setting of cavitary tuberculosis due to the gradual weakening of the adjacent pulmonary artery wall [3]. Pseudoaneurysms are more prone to rupture than true aneurysms because their walls lack the full structural integrity of normal arterial layers and are instead supported by fragile, low-resistance surrounding tissue [4]. The mortality rate of a ruptured PAP is reportedly as high as 50%, highlighting the importance of prompt recognition on radiological scans and subsequent intervention [5].

Lung cancer, particularly adenocarcinoma, is rarely associated with PAPs. However, patients with both lung cancer and PAP carry an even greater risk of hemoptysis as both conditions increase the risk of vascular fragility and bleeding. Hemoptysis occurring in lung cancer is typically attributed to tumor necrosis, the erosion of airways into surrounding vasculature, and neovascularization in and around the tumor [6]. In this case, we describe a pulmonary artery pseudoaneurysm in a patient with stage III lung adenocarcinoma, detected on follow-up imaging despite the absence of hemoptysis or other pulmonary symptoms. The pseudoaneurysm was successfully treated with coil embolization to prevent potential rupture.

## Case presentation

**Figure 1:**
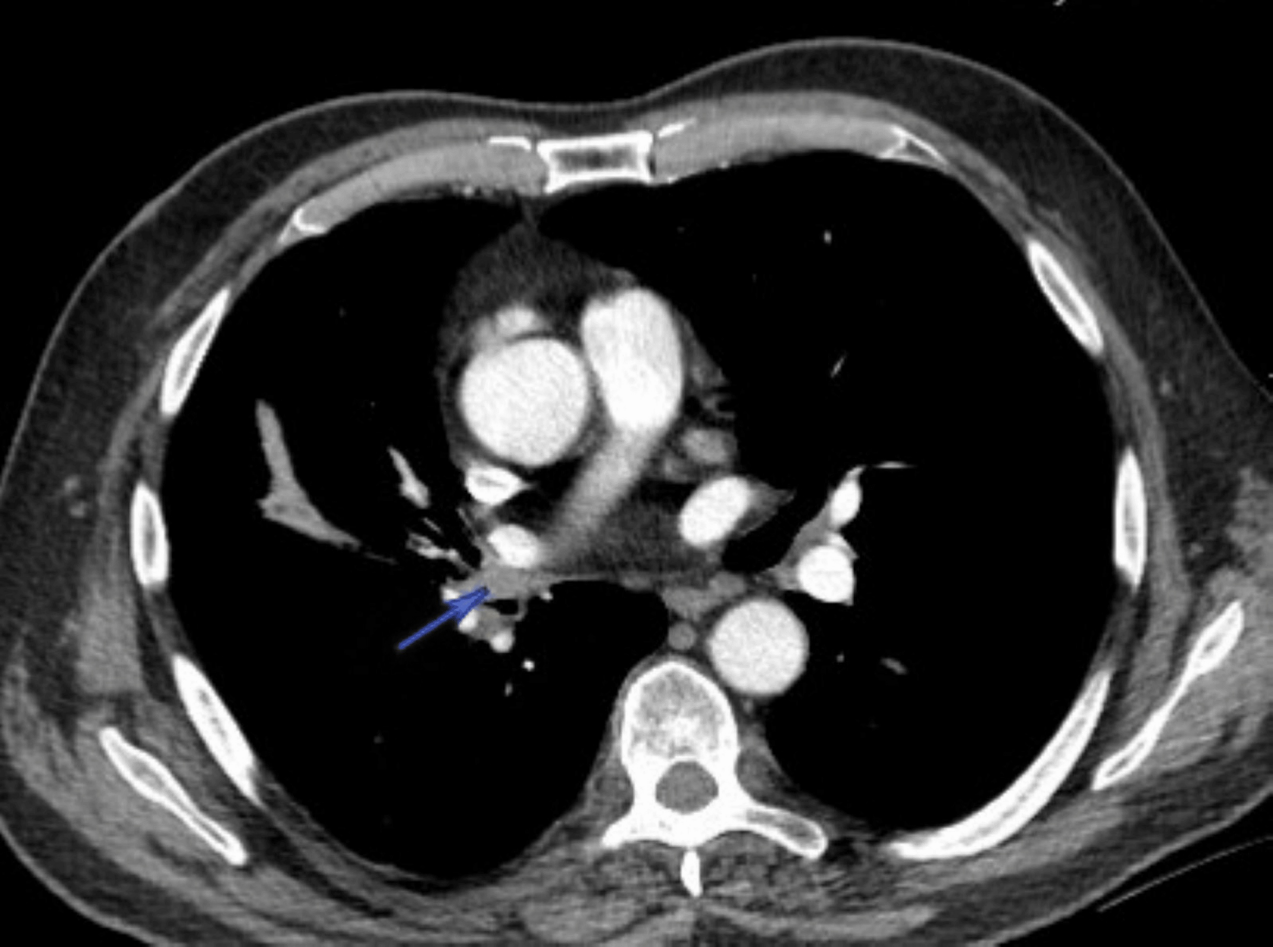
Computed tomography (CT) scan with intravenous (IV) contrast of the chest demonstrating a right hilar nodule (arrow) consistent with non-small cell lung cancer (NSCLC).

A 67-year-old man with a history of stage III non-small cell lung cancer (NSCLC) (T2N2M0) presented for routine oncologic follow-up. He had been diagnosed six months prior, with imaging revealing a 15 × 12 × 22 mm right lower lobe superior segment lung nodule (Figure 1). He had no history of tuberculosis, chronic lung disease, or hemoptysis.

**Figure 2:**
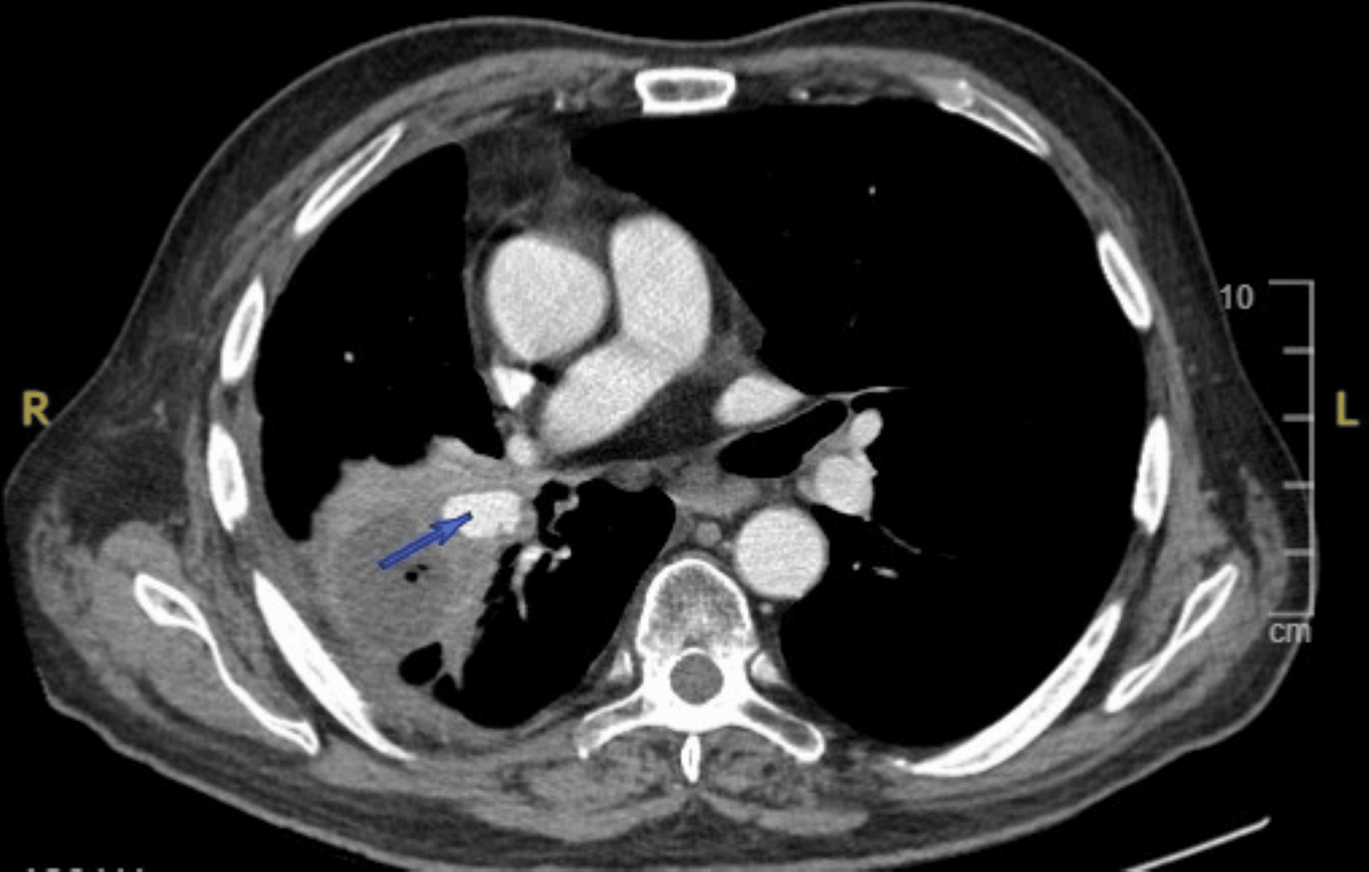
Computed tomography (CT) scan with intravenous (IV) contrast of the chest showing a pulmonary artery pseudoaneurysm (arrow) arising from the right inferior pulmonary artery, adjacent to a necrotic lung tumor.

A routine follow-up chest computed tomography (CT) scan with intravenous (IV) contrast revealed a 23 × 16 × 13 mm pseudoaneurysm arising from the right inferior pulmonary artery, contiguous with pulmonary artery branches in the right lower lobe (Figure 2). Additionally, there was a 43 mm necrotic area in the middle lobe or anterior margin of the right lower lobe, which had expanded compared to prior imaging, raising concern for tumor necrosis. There was no evidence of tuberculous cavitation or infectious process.

The case was discussed at a multidisciplinary cancer conference, where the pseudoaneurysm was noted to be at risk for growth and rupture, despite the patient being asymptomatic. Given the potential for catastrophic hemorrhage, the patient was referred for vascular intervention and underwent successful coil embolization of the pseudoaneurysm, preventing the risk of rupture.

## Discussion

Pulmonary artery pseudoaneurysms (PAPs) are rare vascular abnormalities that carry a high mortality rate if they rupture. While PAPs are most commonly associated with tuberculosis in the form of Rasmussen’s pseudoaneurysm, they can also result from noninfectious causes such as malignancies [7]. Many cases of PAPs are found after the development of massive hemoptysis [8]. However, in this case, the pseudoaneurysm was incidentally detected during a follow-up CT scan for cancer surveillance, leading to prompt discussion, embolization, and the prevention of complications.

Several mechanisms could explain pseudoaneurysm development in lung adenocarcinoma. Tumor necrosis and vascular erosion likely played a significant role in this case. The patient’s CT imaging showed a progressively enlarging necrotic region (43 mm) adjacent to the pseudoaneurysm, suggesting that tumor-induced inflammation weakened the pulmonary artery wall. Another possible explanation is direct tumor infiltration into the pulmonary artery, disrupting the arterial wall and leaving it susceptible to pseudoaneurysm formation. In addition, the patient had undergone radiation therapy to the thoracic region, another potential cause of PAP formation [9].

The management of pulmonary artery pseudoaneurysms requires prompt recognition and intervention. Historically, surgical treatment options of PAPs included pneumonectomy, lobectomy, the interposition of a prosthetic graft or pericardial patch, pulmonary artery ligation, or hilar clamping with arterioplasty [10]. However, today, an endovascular technique is the first-line treatment as it is less invasive and avoids complications of general anesthesia in susceptible patients [11]. In this patient, coil embolization was performed successfully, preventing potential rupture and hemorrhage.

## Conclusions

This case highlights a rare presentation of a pulmonary artery pseudoaneurysm (PAP) in a patient with lung adenocarcinoma, detected incidentally on follow-up imaging. While PAPs are commonly associated with infections such as tuberculosis, they can also arise in malignancy due to tumor necrosis, vascular invasion, or radiation-induced injury. Given the potential for pseudoaneurysm rupture, early detection through imaging and timely intervention are critical in preventing fatal complications.

PAPs should be considered in lung cancer patients, especially when imaging reveals tumor necrosis near vascular structures. Early detection and prompt embolization are crucial to prevent rupture, even in asymptomatic patients.
